# Liposomal Encapsulation of Tartar Emetic Improves Pharmacokinetic Profile and Mitigates Cardiotoxicity in Murine Experimental Model

**DOI:** 10.3390/pharmaceutics17091109

**Published:** 2025-08-26

**Authors:** Larissa Coelho, Marta Aguiar, Mirna Souza, Maria Paiva, Clésia Nascentes, Artur Miranda, Paulo Coelho, Thomas Moore-Morris, Sylvain Richard, Pierre Sicard, Mônica Oliveira

**Affiliations:** 1 Department of Pharmaceutical Products, Faculty of Pharmacy, Universidade Federal de Minas Gerais, Belo Horizonte 31270-901, MG, Brazil; larissadc16@gmail.com (L.C.); martagontijoa@gmail.com (M.A.); 2 Department of Clinical and Toxicological Analysis, Faculty of Pharmacy, Universidade Federal de Minas Gerais, Belo Horizonte 31270-901, MG, Brazil; mirna.dauriol@gmail.com (M.S.); mjnpaiva@yahoo.com.br (M.P.); 3 Department of Chemistry, Instituto de Ciências Exatas, Universidade Federal de Minas Gerais, Belo Horizonte 31270-901, MG, Brazil; clesia@qui.ufmg.br; 4 Department of Physiology and Biophysics, Universidade Federal de Minas Gerais, Belo Horizonte 31270-901, MG, Brazil; santosmiranda.a.edu@gmail.com; 5 René Rachou Institute, Oswaldo Cruz Foundation (FIOCRUZ), Belo Horizonte 30190-009, MG, Brazil; paulo.zech@fiocruz.br; 6 Institut National de la Santé et de la Recherche Médicale (INSERM), Centre National de la Recherche Scientifique (CNRS), Université de Montpellier, 34094 Montpellier, France; thomas.moore-morris@inserm.fr; 7 Physiologie et Médecine Experimentale, Institut National de la Santé et de la Recherche Médicale (INSERM), Centre National de la Recherche Scientifique (CNRS), Université de Montpellier, 34295 Montpellier, France; sylvain.richard@inserm.fr

**Keywords:** liposomes, tartar emetic, cardiotoxicity, echocardiography, pharmacokinetics, antimony, biodistribution

## Abstract

**Background/Objectives:** Visceral leishmaniasis remains a serious public health issue, with current antimonial therapies limited by systemic toxicity. Liposomal drug delivery systems offer a promising strategy to improve therapeutic outcomes. In this context, we previously demonstrated the antileishmanial efficacy of the liposomal formulation containing tartar emetic (Lip-TE) in reducing the parasite load in the spleen and liver of animals experimentally infected with *Leishmania (Leishmania) infantum*, this being an interesting alternative for the treatment of visceral leishmaniasis. In this study, we aimed to evaluate the physicochemical properties, biodistribution, pharmacokinetics, and cardiotoxicity of Lip-TE. **Methods:** Both Lip-Blank and Lip-TE exhibited monodisperse size distributions (PDI < 0.3), with Lip-TE having an increased particle size (205.1 ± 4.5 nm) compared to Lip-Blank (137.5 ± 0.8 nm). Both formulations demonstrated a positive zeta potential of about +17.0 mV. Biodistribution and cardiotoxicity studies were performed using female BALB/c mice, approximately 8 to 10 weeks old. **Results:** A significantly greater splenic, hepatic, and renal accumulation of Lip-TE was observed, particularly at 120 min post-injection, compared to Free TE treatment. The pharmacokinetic profile of the treatment with Lip-TE showed higher systemic exposure (AUC), extended half-life and mean residence time, and reduced clearance compared to Free TE treatment. Echocardiographic analysis revealed that treatment with Free TE significantly reduced the ejection fraction percentage and QRS complex prolongation after 10 days of treatment. These alterations persisted at 30 days, indicating progressive cardiotoxicity. In contrast, Lip-TE treatment preserved cardiac function, revealing the protective effect of the liposomal encapsulation. **Conclusions:** These findings highlight the potential of the Lip-TE treatment to enhance safety and efficacy.

## 1. Introduction

Tartar emetic (TE), or potassium antimony tartrate, is a trivalent antimonial compound historically used in the treatment of parasitic diseases, including leishmaniasis, schistosomiasis, and trypanosomiasis [1,2,3]. The antiparasitic efficacy of TE is well documented; however, its clinical use has been largely abandoned due to its narrow therapeutic window and severe adverse effects, particularly gastrointestinal toxicity and dose-dependent cardiotoxicity [3]. Experimental evidence showed that TE-induced cardiovascular alterations include conduction disturbances and myocardial dysfunction [3], which, together with its rapid systemic clearance, limit its therapeutic potential. Nevertheless, the strong antileishmanial activity of antimonials continues to motivate research into strategies that can improve their safety and efficacy profiles.

Visceral leishmaniasis (VL), also known as kala-azar, is a neglected tropical disease caused by protozoa of the Leishmania donovani and Leishmania infantum complex. It is fatal in more than 95% of untreated cases and remains a serious public health challenge in tropical and subtropical regions, with over 95% of new cases concentrated in a few countries, including Brazil, India, Sudan, and Ethiopia [4,5,6]. The disease primarily affects the liver, spleen, and bone marrow, leading to prolonged fever, weight loss, hepatosplenomegaly, and anemia [6]. Current therapeutic options rely heavily on pentavalent antimonial compounds, amphotericin B formulations, pentamidine, and paromomycin. While effective, these drugs are associated with high toxicity, the need for parenteral administration, and the emergence of resistant Leishmania strains, underscoring the urgent need for safer and more targeted treatments [4,5,7].

Pharmacokinetics and biodistribution studies are essential for evaluating how therapeutic agents, including nanocarriers like liposomes, are absorbed, distributed, metabolized, and excreted (ADME) within the body. Liposomes—spherical vesicles made up of lipid bilayers—have emerged as a promising drug delivery system due to their ability to encapsulate both hydrophilic and hydrophobic drugs, enhance bioavailability, and reduce systemic toxicity [8]. In infectious disease research, liposomal formulations are being investigated for targeted drug delivery, especially for conditions such as leishmaniasis, where they improve therapeutic outcomes by increasing drug bioavailability while minimizing toxicity [9].

Previous studies conducted by our research group showed the effectiveness of liposomes containing TE (Lip-TE) in reducing parasite load in liver and spleen, and acute toxicity in the treatment of leishmaniasis [10]. In order to ensure the application of this formulation, it is crucial to have a detailed understanding of the pharmacokinetics behavior, biodistribution, and toxicity, particularly regarding its tissue-specific accumulation and clearance pathways, as well as the cardiotoxicity induced by the TE treatment. Therefore, in the studies carried out, we were interested in performing biodistribution and pharmacokinetic analyses of the treatments of mice with Free TE and Lip-TE, using advanced analytical chemistry techniques, which provide crucial insights into the behavior of these formulations in vivo [9,11]. For these studies, we used nitric acid digestion followed by atomic absorption spectrometry in a graphite furnace, which is capable of accurately quantifying the drug accumulation in various organs and plasma. This allows for an accurate evaluation of metal-based drug carriers, as demonstrated in studies of validation of a graphite furnace atomic absorption spectrometry method for determining total antimony in organs and plasma from animal models [11,12].

Furthermore, the cardiotoxic effect of TE is well described in the literature [13], and it is also extremely important to investigate the existence of cardiac effects after treatment of mice with Lip-TE. Echocardiography (ECHO) and electrocardiography (ECG) are two key imaging modalities to assess cardiac structure and function. These approaches provide essential insights into myocardial function and electrical activity, respectively. ECHO is a non-invasive ultrasound technique widely used to evaluate myocardial structure, contractility, and hemodynamics. It plays a crucial role in diagnosing and monitoring cardiovascular diseases [14]. Differently, ECG records the heart’s electrical impulses, which helps in detecting arrhythmias, ischemic events, and conduction abnormalities [15]. When used together, these techniques offer a comprehensive evaluation of cardiac physiology in both clinical and preclinical settings.

Thus, this article aims to explore the integration of biodistribution, pharmacokinetics, and ECHO and ECG studies, which are particularly important in the preclinical evaluation of the treatment of visceral leishmaniasis using Lip-TE.

## 2. Materials and Methods

### 2.1. Materials

Potassium antimony tartrate trihydrate (tartar emetic—TE) (Sigma-Aldrich, St. Louis, MO, USA), sodium chloride (Merck, Darmstadt, Germany), egg phosphatidylcholine (EPC) (Lipoid GmbH, Ludwigshafen, Germany), 3ß-[N-(N′,N′-dimethylaminoethane)-carbamoyl]cholesterol (DC-Chol) (Chem-Impex International, Inc., Wood Dale, IL, USA), chloroform (Vetec Quimica Fina Ltd.a., Duque de Caxias, Brazil), dibasic sodium phosphate heptahydrate (Merck, Darmstadt, Germany), anhydrous monobasic potassium phosphate (Vetec Quimica Fina Ltd.a., Duque de Caxias, Brazil), HEPES (Vetec Quimica Fina Ltd.a., Duque de Caxias, Brazil), ethylenediaminetetraacetic acid disodium salt (EDTA-Na_2_) (Ecibra, São Paulo, Brazil), Dulbecco’s Phosphate Buffered Saline (DPBS) (Sigma-Aldrich, Gillingham, UK). For PBS preparation, the following reagents were used: anhydrous monobasic potassium phosphate, dibasic sodium phosphate heptahydrate, and sodium chloride.

For Sb quantification using graphite furnace atomic absorption spectrometry (GFAAS) (Agilent Technologies, model 240Z AA—200 Series AA, Santa Clara, CA, USA), all reagents were of analytical grade: 65% (*v*/*v*) nitric acid (Merck, Darmstadt, Germany), 30% (*v*/*v*) hydrogen peroxide (Labsynth, Diadema, Brazil), 1000 mg L^−1^ antimony reference solution (Merck, Darmstadt, Germany), zirconium (Merck, Darmstadt, Germany), and Triton X-100 (Merck, Darmstadt, Germany).

### 2.2. Liposome Preparation

Liposome preparation followed the same methodology, as described previously [10], using the reverse-phase evaporation method. Briefly, EPC and DC-Chol (20 mM total lipid concentration, 7:3 molar ratio, respectively) were dissolved in chloroform, and the solvent was removed under reduced pressure to form lipid films for both Lip-TE and blank liposomes. The lipids were then redissolved in diethyl ether, and TE solution (60 g/L in PBS) was added. The mixture underwent solvent removal via rotary evaporation, and the resulting liposomes were size-calibrated by extrusion through polycarbonate membranes (0.4 µm for 10 cycles, 0.2 µm for 5 cycles). Non-encapsulated TE was eliminated by ultracentrifugation at 150,000× *g*, 4 °C, for 120 min, followed by resuspension in PBS (pH 7.4).

Liposome characterization included size and polydispersity index (PDI) analysis using dynamic light scattering (DLS) at 25 °C and a 90° angle, while electrophoretic mobility measurements determined the zeta potential (Zetasizer NanoZS90, Malvern Instruments, Malvern, UK).

#### Drug Quantification

A previously validated protocol was employed to determine the encapsulation content (EC) of TE. Quantification of TE was performed using graphite furnace atomic absorption spectrometry (GFAAS; Agilent Technologies, 240Z AA—200 Series AA, Santa Clara, CA, USA). Briefly, aliquots collected before and after ultracentrifugation were diluted to a theoretical final concentration of 50 µg/L in ultra-pure water containing 0.5% (*v*/*v*) HNO_3_. Sb concentrations were measured under pyrolysis and atomization temperatures of 700 °C and 2000 °C, respectively.

### 2.3. Biodistribution and Pharmacokinetic Studies

**Figure 1:**
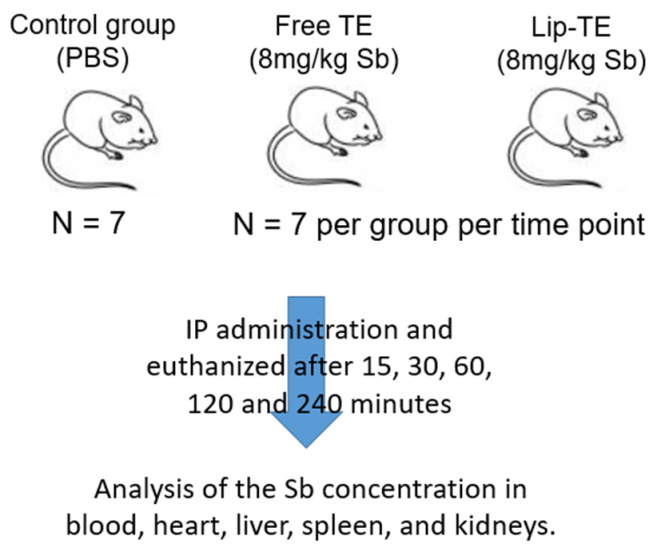
Diagram summarizing pharmacokinetic and biodistribution studies.

In order to evaluate the biodistribution and pharmacokinetics of the Lip-TE treatment, female BALB/c mice (8 weeks old, 18–22 g) were used. The studies were approved by the Ethics Committee on Animal Use of the Federal University of Minas Gerais (CEUA/UFMG) under protocols n^o^ 240/2018 and 49/2024. The animals were weighed prior to treatment initiation and randomly assigned to three groups (n = 7 per group per time point): PBS (control group), Free TE, and Lip-TE. The solution containing Free TE was prepared by dissolution of 67 mg of TE in 30 mL of PBS. Thus, the final concentration of the TE solution was 2.2 g/L. Each group received an intraperitoneal dose (0.2 mL) of Sb at 8 mg/kg. The animals were euthanized at predetermined time points of 15, 30, 60, 120, and 240 min post-administration, as illustrated in Figure 1.

To collect blood samples, the animals were anesthetized with a mixture of ketamine (80 mg/kg) and xylazine (15 mg/kg). Blood was then collected into tubes containing 0.1 M EDTA. Following blood collection, the animals were euthanized, and organs were harvested. The organs were collected and prepared as follows: 1000 mg of heart, 100 mg of liver, 1000 mg of spleen, and 500 mg of kidney samples in PBS were digested with nitric acid. Following digestion, the samples were transferred to 25 mL volumetric flasks and diluted with deionized water. For plasma analysis, 25 µL of each sample was used. The PBS group served as a control to generate calibration curves fortified with antimony.

Absorbance measurements were conducted using an atomic absorption spectrometer (Agilent Technologies, model 240Z AA—200 Series AA, Santa Clara, CA, USA) operated via SpectrAA software version 5.2 Pro. The thermal program for total Sb determination in different organs and plasma, encompassing pre-drying, drying, pyrolysis, atomization, and cleaning stages, is detailed in Table 1.

Pharmacokinetic parameters, including C_max_ (maximum plasma concentration), t_1/2_ (half-life), AUC (area under the concentration–time curve), MRT (mean residence time), and Cl (clearance), were analyzed to assess drug disposition. The pharmacokinetic calculations were performed using PKSolver 2.0, a validated Excel-based software, using non-compartmental analyses and the linear trapezoidal method [16]. Additionally, standard pharmacokinetic principles and methodologies were referenced from the established literature [17].

### 2.4. In Vivo Cardiotoxic Assessment

In order to evaluate cardiotoxicity in vivo, a study was performed on female BALB/c mice (8–10 weeks old, 20–25 g). The study was approved by the ethics committee of the Université de Montpellier (CCEA n° 36) under protocol #46300-2023121510104831 v4.

**Figure 2:**
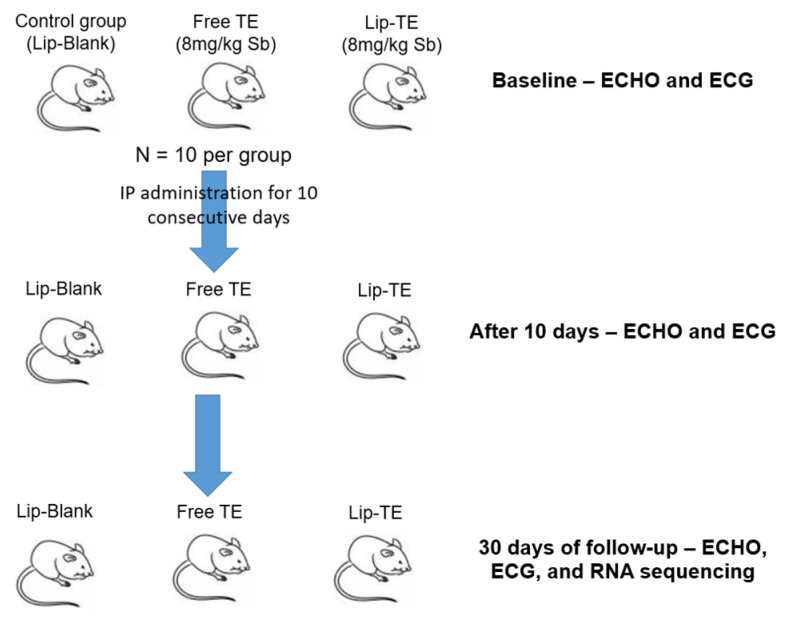
Diagram summarizing cardiotoxic assessment.

The animals were randomly divided into three treatment groups (*n* = 10 per group): Free TE, Lip-TE, and blank liposomes (Lip-Blank) (Figure 2). BALB/c mice received repeated intraperitoneal (IP) injections (0.2 mL) of Sb at a dose of 8 mg/kg/day for 10 consecutive days, resulting in a cumulative dose of 80 mg/kg of Sb. Throughout the experimental period, body weight was individually monitored as an indicator of systemic toxicity. All animals were weighed at baseline (prior to the first injection) and periodically throughout the 30-day follow-up. Weight variation was calculated as the percentage change from the baseline weight to the final measurement on day 30, using the following formula: %Δ weight = [(weight day 30 − weight day 0)/weight day 0] × 100. This approach enabled the assessment of both acute and delayed treatment-related effects on animal health and nutritional status.

#### 2.4.1. Cardiac Function and Electrophysiology Assessments

Cardiac function and electrophysiological parameters were evaluated using ECG and ECHO before treatment (baseline), one day after final administration, and at 30 days of follow-up. High-resolution ECHO was performed using a Vevo 3100 system (Fujifilm VisualSonics, Toronto, ON, Canada) equipped with a 40 MHz MX550D ultrasound probe to assess left ventricular (LV) function, including ejection fraction and longitudinal strain analysis for cardiac deformation, following the American Physiological Society guidelines [18].

ECHO was conducted under general anesthesia with inhaled isoflurane (2.5%), while ECG signals, respiratory rate, and body temperature were continuously monitored. Left ventricular wall thickness and diameter were measured at the level of the papillary muscles using a two-dimensional parasternal long-axis view in M-mode. The left ventricular ejection fraction (LVEF) and relative wall thickness were calculated. Endocardial end-diastolic and end-systolic areas were traced, and LV volumes were determined using the Simpson disk method, with LVEF calculated as follows:

$$LVEF(\%)=\frac{LVEDv-LVESv}{LVEDv}\times 100$$

LVEDv refers to the left ventricular end-diastolic volume, measured when the ventricle is maximally filled. LVESv refers to the left ventricular end-systolic volume, measured at the end of ventricular contraction. LVEF represents the proportion of blood ejected from the left ventricle during each cardiac cycle, expressed as a percentage.

Global and regional longitudinal strain analysis were performed using a parasternal long-axis view [18]. Offline image analysis was performed using VisualSonics VevoLab 3.1.0 and VevoStrain 2.0 software.

Lead II was used for measurement of ECG recordings and was continuously acquired for 5 min using subcutaneous needle electrodes connected to shielded cables while mice were under isoflurane anesthesia (2.5%) with spontaneous respiration. Ten complexes were selected, and the resulting waveform was manually analyzed using LabChart7 software (ADInstruments, Sydney, Australia). The isoelectric baseline was defined as the segment between the end of the T wave and the start of the P wave. ECG intervals, including RR, PR, QRS, QT, and QTc, were calculated [19].

#### 2.4.2. RNA Extraction and Sequencing

Following cardiac function and electrophysiological assessments, all animals (n = 30) were euthanized using 2.5% isoflurane, followed by cervical dislocation. Hearts were collected, and the lower portion (apex) was used for RNA extraction and subsequent RNA sequencing. Total RNA was extracted using the Quick-DNA/RNA™ Miniprep Kit (Zymo Research, Irvine, CA, USA), following the manufacturer’s protocol.

The purified RNA was used to generate cDNA libraries with the SMART-Seq^®^ Stranded Kit (Takara Bio, San Jose, CA, USA), ensuring high-quality transcripts for sequencing. The key steps included first-strand cDNA synthesis; amplification and adapter addition; ribosomal RNA depletion; and second PCR amplification. The final cDNA libraries were validated for fragment size and concentration using a Fragment Analyzer (High and Standard Sensitivity NGS kits, Agilent Technologies, Santa Clara, CA, USA) and quantified via qPCR using a Roche LightCycler^®^ 480 Instrument (Roche Diagnostics, Mannheim, Germany).

For statistical analysis, the R packages EdgeR 4.7 [20,21] and DESeq2 1.4 [22] were used to identify differentially expressed genes. Both tools employ a negative binomial distribution to model count data and utilize a generalized linear model (GLM) framework. Tests performed for this experiment consist of 3 different treatments (Lip-TE, Free TE, Lip-Blank), each with 5 replicates. The following comparisons were made: Free TE vs. Lip-Blank, Lip-TE vs. Free TE, and Lip-TE vs. Lip-Blank.

### 2.5. Statistical Analysis

Statistical analyses for studies were conducted using GraphPad Prism 8.0 software. The normality of the data was assessed using the Shapiro–Wilk test. Differences between experimental groups were evaluated through hypothesis testing, employing Student’s *t*-test for pairwise comparisons and one-way or two-way analysis of variance (ANOVA) for multiple group comparisons, followed by Tukey’s post hoc test. A 95% confidence interval was applied in all analyses, and statistical significance was defined as *p* < 0.05.

## 3. Results

### 3.1. Physicochemical Characterization of Liposomes

The physicochemical characterization of the liposomal formulation is essential for ensuring its safety, stability, and therapeutic effectiveness, especially when it is intended for parenteral administration. Key parameters to consider include vesicle size, PDI, and zeta potential, which are important indicators of the homogeneity, colloidal stability, and surface charge of liposomal systems. These factors directly influence its biodistribution and cellular uptake profiles [23].

As shown in Table 2, both Lip-Blank and Lip-TE formulations demonstrated monodisperse size distributions, with a PDI below 0.3, indicating uniformity of the formulation and low aggregation tendencies. As previously reported in our studies [10], Lip-Blank exhibited a smaller average particle size (137.5 ± 0.8 nm) compared to Lip-TE (205.1 ± 4.5 nm). This size increase is expected due to the incorporation of the therapeutic agent TE into the liposomal core.

The positive zeta potential observed in both formulations (+17.0 mV) can be attributed to the inclusion of the cationic lipid DC-Chol into the bilayer of the liposomes.

The encapsulation efficiency, represented here as the encapsulation content (2.22 ± 0.38 g/L) for Lip-TE, demonstrates successful loading of TE into the liposomal formulation.

### 3.2. Biodistribution Study

**Figure 3:**
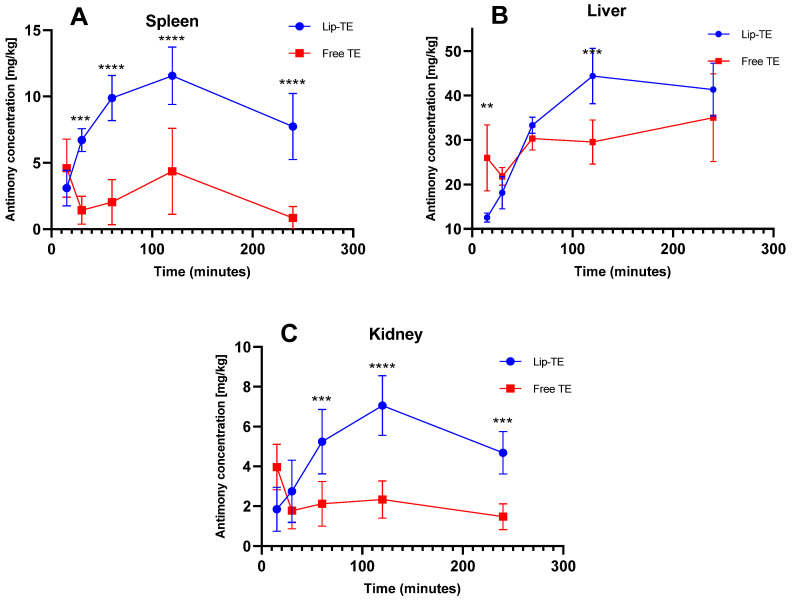
Sb concentrations in (**A**) spleen, (**B**) liver, and (**C**) kidney following single intraperitoneal injection of Lip-TE or Free TE (8 mg/kg Sb^3+^) at 15, 30, 60, 120, and 240 min. Data are mean ± standard deviation. ** *p* < 0.01, *** *p* < 0.001, **** *p* < 0.0001 for differences between treatments at the same time point. Shapiro–Wilk test confirmed normality. One-way ANOVA and Tukey’s post hoc test were applied. n = 7 per group per time point.

The study of the biodistribution of bioactive substances is crucial for evaluating both therapeutic efficacy and potential toxicity of the treatment. GFAAS was employed due to its high sensitivity and low sample consumption, making it ideal for quantifying trace antimony concentrations in various tissues following administration [24,25,26]. It is worth highlighting that no significant differences (*p* > 0.05) in body weight were noted between the treatment groups, ruling out that parameter as a confounding factor. Figure 3 illustrates the time-dependent Sb accumulation in spleen (A), liver (B), and kidney (C) after a single intraperitoneal dose of Lip-TE or Free TE (8 mg/kg Sb^3+^). Heart data could not be presented, as Sb concentrations were consistently below the analytical limit of detection (LoD).

A significantly higher Sb concentration in the spleen was observed at almost all time points after administration of the Lip-TE treatment compared to the Free TE treatment (Figure 3A). The peak of greatest antimony accumulation occurred at 120 min, followed by a gradual decline up to 240 min. This pattern is consistent with the mononuclear phagocyte system (MPS) uptake, where liposomal carriers are preferentially taken up by splenic macrophages [23]. In contrast, the Free TE treatment showed lower and flatter accumulation, suggesting reduced uptake or faster clearance. In the liver, a higher Sb accumulation also occurred after injection of the Lip-TE treatment at 120 min (Figure 3B). The liver is a major MPS organ, and this enhanced hepatic accumulation of Lip-TE supports the role of liposomal carriers in hepatotropic distribution, as we already observed in our previous studies showing a greater efficacy of leishmaniasis treatment with a lower parasitic load in the liver and spleen [10]. Notably, Free TE treatment showed lower hepatic accumulation than Lip-TE treatment.

In the kidney, a pronounced amount of Sb was detected for the Lip-TE-treated group, presenting also a maximum peak at 120 min followed by a gradual decline up to 240 min (Figure 3C). In contrast, the Free TE-treated group showed an accumulation of Sb in the kidney that was significantly lower and relatively constant. This enhanced renal uptake for Lip-TE-treated mice may be due to secondary clearance pathways for the liposomal formulation or slow release of Sb from the nanocarrier followed by renal filtration.

### 3.3. Pharmacokinetic Study

The systemic behavior of the Free TE and Lip-TE treatments was investigated by monitoring the plasma concentration of Sb at various time points post-injection; the pharmacokinetic (PK) parameters are summarized in Table 3. 

Following administration, the maximum plasma concentrations (C_max_) of Free TE and Lip-TE treatments were 1.7 ± 0.3 mg/L and 1.3 ± 0.2 mg/L, respectively. Although the C_max_ values were comparable, notable differences were observed in other PK parameters. The elimination half-life (t_1/2_) for Lip-TE treatment (461.9 ± 76.9 min) was significantly prolonged compared to Free TE treatment (177.5 ± 30.8 min), indicating a slower elimination rate of the liposomal formulation.

In terms of systemic exposure, the area under the plasma concentration–time curve extrapolated to infinity (AUC_0–inf_) was approximately threefold higher for Lip-TE treatment (675.5 ± 50.8 mg/L·min) compared to Free TE treatment (210.5 ± 24.1 mg/L·min), also reflecting a significantly enhanced drug retention in circulation (*p* < 0.05). The AUC from time zero to the last measurable concentration (AUC_0–t_) showed a similar trend, with values of 213.9 ± 29.2 mg/L·min for Lip-TE treatment and 110.3 ± 8.1 mg/L·min for Free TE treatment.

The mean residence time (MRT_0–inf_) was slightly longer for Lip-TE treatment (296.4 ± 43.1 min) than for Free TE treatment (253.0 ± 40.3 min), although this difference was not statistically significant. Furthermore, the apparent volume of distribution during the terminal phase (Vz) was reduced in the Lip-TE-treated group (8.7 ± 1.3 vs. 13.3 ± 2.8 (mg/kg)/(mg/L)), suggesting more limited tissue distribution. Finally, the clearance rate (Cl) of the Lip-TE treatment (0.01 ± 0.001 (mg/kg)/(mg/L)/min) was significantly lower than that of Free TE treatment (0.04 ± 0.003 (mg/kg)/(mg/L)/min), further supporting the prolonged circulation time and slower elimination profile of the liposomal formulation (*p* < 0.05).

### 3.4. Evaluation of the Left Ventricular Function and Atrial Remodeling in BALB/c Mice Using High-Resolution Echocardiography

The acquired images allowed for the quantification and analysis of several key cardiac parameters, including heart rate (HR), ejection fraction (EF%), cardiac output (CO), stroke volume (SV), systolic diameter (Diameter S), and diastolic diameter (Diameter D), among others. Throughout the procedure, respiratory rate and body temperature were monitored and remained within physiological limits, with a temperature ranging from 36 to 37 °C, and the respiratory rate was between 145 and 170 breaths per minute.

#### 3.4.1. Comparison of the Cardiac Function Between Lip-Blank, Lip-TE, and Free TE Treatments

Cardiac function and structural integrity were assessed in BALB/c mice treated with Lip-Blank (control), Lip-TE (8 mg/kg of Sb), or Free TE (8 mg/kg of Sb) over a 30-day monitoring period. Echocardiographic parameters such as EF%, longitudinal strain, and others were used to evaluate potential cardiotoxic effects of the TE-containing formulations administered.

**Figure 4:**
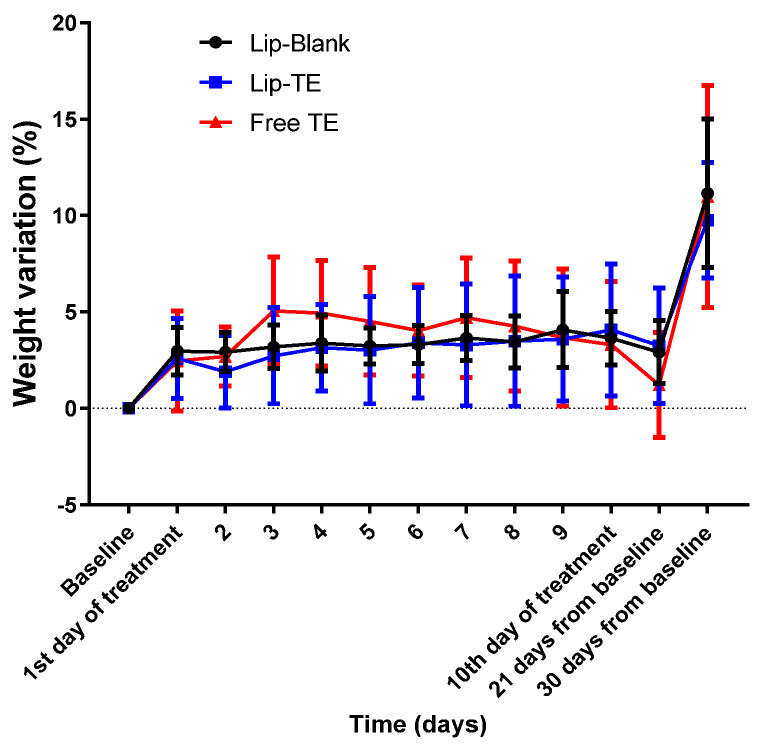
Body weight variations observed during 30 days after 10-day intraperitoneal administration of Lip-Blank (control), Lip-TE (8 mg/kg Sb), and Free TE (8 mg/kg Sb). Data are presented as mean ± standard deviation. Sample size per group: n = 10.

Echocardiographic analysis revealed significant impairments in cardiac function of the Free TE-treated group, and systemic toxicity indicators such as body weight variation were closely monitored to rule out generalized toxicity effects. Weight monitoring in preclinical cardiotoxicity studies is essential since cardiac dysfunction can often be accompanied by cachexia or metabolic alterations [27]. However, in this study, the lack of substantial weight loss reinforces that the cardiac dysfunction observed in the Free TE-treated group was not secondary to malnutrition, dehydration, or other systemic complications but rather a direct effect of the treatment with Free TE on myocardial tissue (Figure 4).

As depicted in Figure 4, body weight remained stable across all treatment groups, with no weight loss greater than 15% throughout the experimental period. This finding is critical, as body weight loss beyond 15% is often indicative of systemic toxicity or severe adverse effects [28]. The slight increase in weight toward the end of the monitoring period could be attributed to natural growth and metabolic adjustments. Importantly, the absence of drastic weight drops suggests that neither Lip-TE treatment nor Free TE treatment induced significant systemic distress.

EF% is a useful measure of LV systolic function, indicating the heart’s ability to pump blood efficiently during each contraction. A decrease in the EF% value is often associated with cardiac dysfunction, contractile impairment, or cardiomyopathy [29]. Longitudinal strain is an essential echocardiographic parameter for detecting subclinical myocardial dysfunction, often preceding reductions in the EF%. A decline in strain indicates deterioration of the myocardial contractility, which may arise due to cytotoxic damage, fibrosis, or increased myocardial stiffness [18].

**Figure 5:**
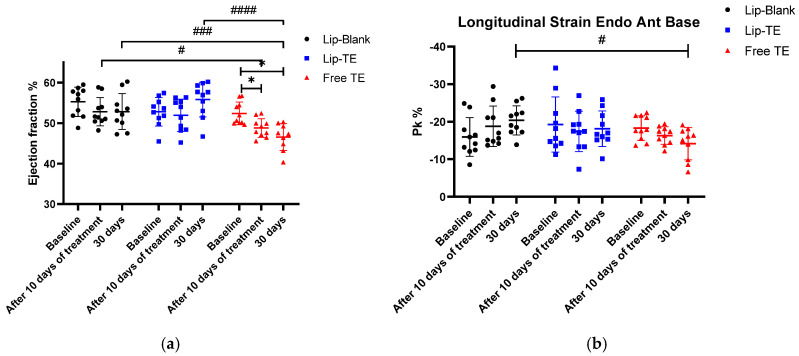
Ejection fraction (%) (**a**), and segmental longitudinal strain (%) (**b**), measured after 10-day intraperitoneal administration of Lip-Blank, Lip-TE (8 mg/kg of Sb), and Free TE solutions (8 mg/kg of Sb), with 30-day follow-up. Data are expressed as mean ± standard deviation. * Indicates *p* < 0.05 within the same group across time points. # indicates *p* < 0.05, ### indicates *p* < 0.001, and #### indicates *p* < 0.0001, comparing the Lip-Blank and Lip-TE groups with the Free TE group after 10 days of treatment and 30 days of follow-up. Normality confirmed via the Shapiro–Wilk test. Two-way ANOVA followed by Tukey’s post hoc test was used. n = 10 per group.

As depicted in Figure 5a, a significant reduction in the EF% was observed exclusively in the Free TE-treated group after 30 days of follow-up. This decline suggests a direct cardiotoxic effect of the Free TE treatment, impairing myocardial contractile function. Importantly, the Lip-TE-treated group maintained EF% levels comparable to the control (Lip-Blank) group, indicating a potential protective role of the liposomal formulation against TE-induced toxicity. The statistical analysis confirms a significant intra-group decrease in the EF% values within the Free TE-treated group (*p* < 0.05). This decline after 30 days suggests that prolonged exposure to Free TE treatment leads to progressive deterioration of the systolic function. Conversely, the Lip-TE and control-treated groups exhibited stable EF% values throughout the study, further indicating that the cardiotoxic effects were specific to the Free TE formulation. As depicted in Figure 5b, the Free TE-treated group showed a significant reduction in the longitudinal strain (%) after 30 days (*p* < 0.05 vs. the control group). This impairment was not observed in the Lip-TE- or Lip-Blank-treated groups, suggesting a direct cardiotoxic effect of Free TE.

ECG analysis is a fundamental tool in preclinical research to evaluate cardiac electrophysiological alterations induced by pharmacological interventions. The ECG provides valuable insights into conduction velocity, repolarization abnormalities, and arrhythmic risk, making it an essential parameter for assessing cardiotoxic effects [15]. In the present study, we investigated the effects of the TE solution on cardiac conduction over time, focusing on changes in key ECG parameters.

**Figure 6:**
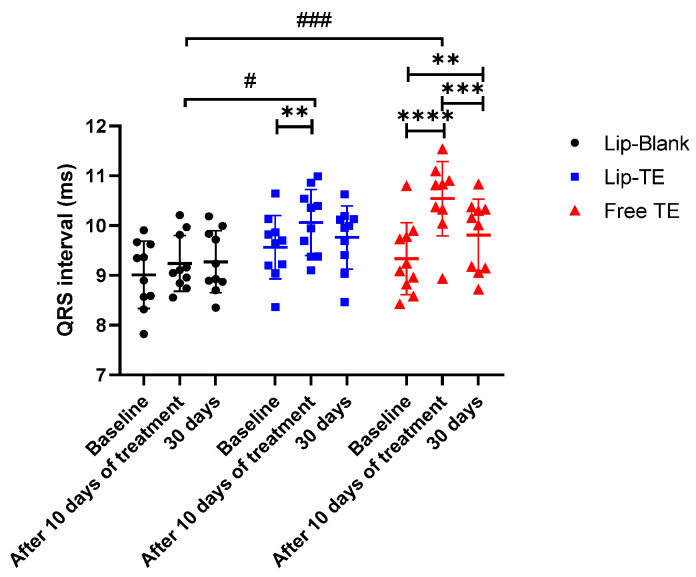
QRS interval after 10-day administration of Lip-Blank, Lip-TE (8 mg/kg Sb), and Free TE solutions (8 mg/kg Sb), followed by 30 days of monitoring. Data are mean ± standard deviation. ** *p* < 0.01, *** *p* < 0.001, **** *p* < 0.0001 for within-group differences. # *p* < 0.05, ### *p* < 0.001 for between-group comparisons at the same time point. Normality confirmed using the Shapiro–Wilk test. Analysis was performed using two-way ANOVA followed by Tukey’s test. n = 10.

After 10 days of Free TE treatment, we observed a significant prolongation of the QRS interval, which persisted at 30 days, as shown in Figure 6. The increase in QRS duration suggests that TE may be affecting ventricular conduction pathways, possibly through structural remodeling or direct cardiotoxic effects. In the case of Lip-TE-treated mice, an increase in the QRS interval was observed after 10 days of administration, which returned to the baseline value after 30 days of injection. It is interesting to note that a statistical difference was observed between the Lip-Blank- and Lip-TE-treated groups, but it was not as pronounced as when compared with the Free TE-treated group.

**Figure 7:**
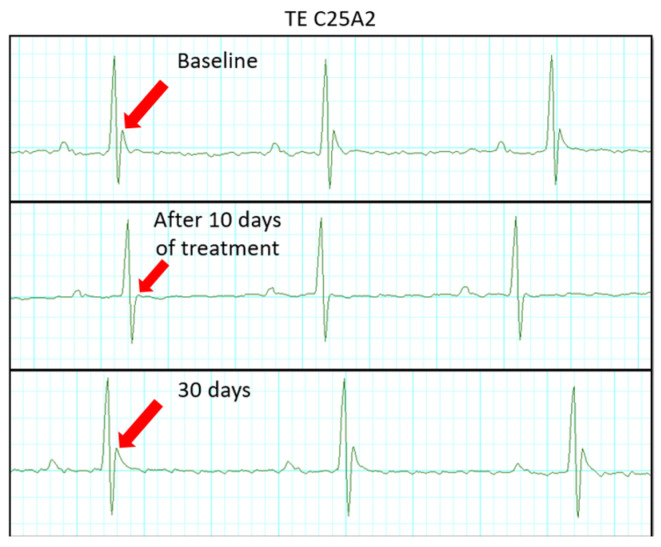
Representative ECG recordings from an animal treated with Free TE at different time points (baseline, 10 days of treatment, and 30 days). J wave signalized by red arrows.

Figure 7 presents representative ECG recordings from an animal treated with Free TE at three distinct time points: baseline (pre-treatment), after 10 days of treatment, and after 30 days. These traces reveal notable changes in cardiac electrical activity, particularly in the QRS interval and J wave morphology. Interestingly, the J wave, which was present at baseline, was no longer visible after 10 days of Free TE treatment, indicating a transient alteration in ventricular repolarization. However, the J wave reappeared after 30 days of follow-up, suggesting partial recovery of the electrophysiological pattern over time. Notably, such disappearance and reappearance of the J wave were not observed in the group treated with Lip-TE, in which the J wave morphology remained stable throughout the treatment period, indicating a more favorable cardiac safety profile.

The persistence of an increased QRS interval at 30 days suggests progressive electrical and structural remodeling of the myocardium, which could correlate with the decrease in the EF% observed in echocardiographic analysis (Figure 5). The persistence of these ECG changes at 30 days suggests that Free TE treatment induces sustained electrophysiological alterations, which may underlie the observed decline in cardiac performance.

#### 3.4.2. RNA Sequencing

**Figure 8:**
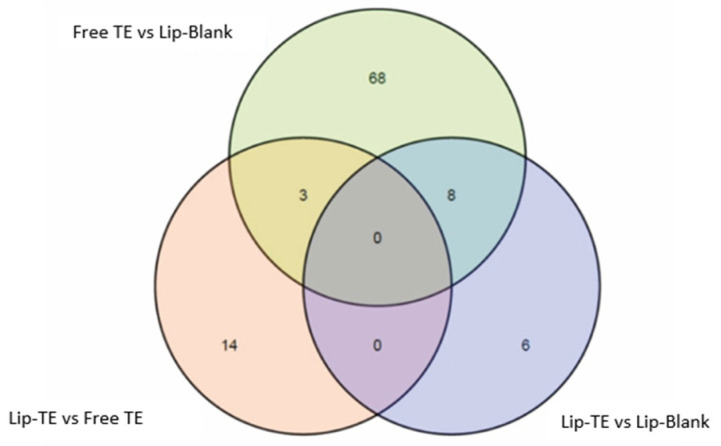
Venn diagram showing the number of DEGs identified by either EdgeR or DESeq2 in each pairwise comparison. Numbers represent the union of DEGs obtained from both tools.

RNA sequencing showed differential expression among groups, with pairwise comparisons revealing that the highest number of differentially expressed genes (DEGs) was observed between the Free TE and Lip-Blank groups (Figure 8, Table 4). Interestingly, the use of liposomes was associated with a lower expression of several markers of TE-induced adverse remodeling.

**Figure 9:**
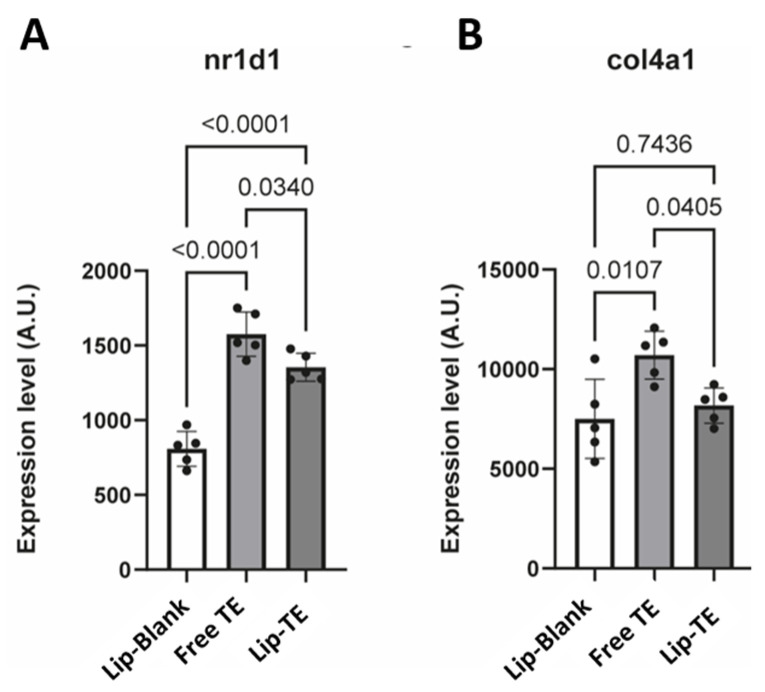
Effects of Free TE and Lip-TE treatments on gene expression levels of *NR1D1* (**A**) and *Col4a1* (**B**), determined by RNA sequencing. Data are presented as mean ± standard deviation (n = 5). Normality confirmed using the Shapiro–Wilk test. Statistical comparisons among groups were performed using one-way ANOVA followed by Tukey’s multiple comparisons test. *p*-Values < 0.05 were considered statistically significant.

Notably, nuclear receptor subfamily 1 group D member 1 (*NR1D1*) was significantly upregulated in the Free TE group compared to the Lip-TE and Lip-Blank groups (Figure 9A). Increased *NR1D1* expression has been previously associated with adverse vascular remodeling, notably of the aorta [30]. Furthermore, genes involved in crosslinking of collagen fibrils (*col4a1*, *pxdn*) were expressed at a higher level in Free TE hearts as compared to Lip-Blank and Lip-TE hearts (Figure 9B). Altogether, these results suggest that the use of liposomes protects the myocardium from TE-induced toxicity.

## 4. Discussion

This study provides a comprehensive assessment of the Lip-TE treatment in terms of pharmacokinetics, biodistribution, and cardiotoxicity using an in vivo murine model. The results demonstrate that liposomal encapsulation of TE offers significant therapeutic and safety advantages compared to the free drug. This discussion integrates the key findings across analytical, pharmacological, and physiological parameters to contextualize the benefits of the use of Lip-TE.

The increase in the vesicle diameter upon drug loading is consistent with literature findings, where incorporation of hydrophilic drugs into liposomes leads to expansion of internal aqueous volume or bilayer reorganization to accommodate the payload [8,23]. Moreover, the observed cationic surface charge may contribute to colloidal stability via electrostatic repulsion and also enhance interaction with negatively charged cell membranes, particularly macrophages, which are central to Leishmania infection [31,32]. The particle size of both formulations (<250 nm) is within the optimal range for passive targeting of the mononuclear phagocyte system (MPS), facilitating enhanced uptake by liver and spleen macrophages through the enhanced permeability and retention (EPR) effect and receptor-mediated endocytosis [23]. This organotropism is crucial for treating visceral leishmaniasis, as these organs harbor the parasite reservoir [10,33].

Biodistribution analysis revealed significantly higher accumulation of Sb in the spleen, liver, and kidneys following Lip-TE treatment compared to Free TE treatment. This preferential uptake by MPS-related organs aligns with the expected behavior of liposomal carriers and supports their role in targeted drug delivery [32]. Importantly, the concentration of Sb in the heart remained below the detection limit in Lip-TE-treated animals, suggesting a lower risk of direct myocardial exposure. This is particularly relevant considering the known cardiotoxicity associated with prolonged or high-dose antimonial therapies. The findings also reinforce the utility of GFAAS for accurate quantification of metals in tissues and highlight the ability of delivery systems to modulate tissue-specific drug exposure [11].

Pharmacokinetic profiling showed that Lip-TE treatment significantly altered systemic drug behavior compared to Free TE treatment. Key parameters such as reduced C_max_, and increased half-life and AUC indicate prolonged circulation and controlled drug release. These characteristics are hallmarks of nanocarrier systems, which protect drugs from rapid metabolism and clearance, ensuring more stable plasma concentrations and longer therapeutic windows [23,31]. The over three-fold increase in AUC_0–inf_ observed with Lip-TE treatment supports its ability to sustain effective plasma levels, a critical factor in treating chronic parasitic infections. While it is generally expected that MRT exceeds t_1/2_, this is not a strict rule. Particularly in formulations with altered pharmacokinetic profiles—such as liposomal or nanoparticle-based drug delivery systems—variations in absorption, distribution, and clearance may result in t_1/2_ and MRT values that are of similar magnitude or even inverted. For example, Reddy and Murthy (2004) demonstrated that for doxorubicin-loaded poly(butyl cyanoacrylate) nanoparticles administered intraperitoneally, t_1/2_ exceeded MRT in some cases, despite a prolonged systemic exposure, due to differences in formulation behavior and tissue distribution between nanoparticle types (e.g., dispersion vs. emulsion polymerization) [34]. Similarly, nanoparticle formulations often exhibit complex release kinetics and reduced tissue distribution that may affect MRT independently of terminal elimination rates, as also highlighted by Lages et al. (2021) in the context of PEGylated nanoparticles [35].

It is also essential to consider the impact of the IP administration route on these parameters. The slower clearance and reduced volume of distribution observed with Lip-TE treatment could be attributed not only to liposomal encapsulation but also to retention of the formulation in the peritoneal cavity before systemic absorption [31,36]. Therefore, two hypotheses may explain the different pharmacokinetic profile between the two treatments: (i) delayed systemic entry from the peritoneum and (ii) prolonged circulation due to liposomal encapsulation. Both factors likely contribute to the observed systemic behavior.

The cardiotoxic effects of Free TE treatment were clearly reflected in the echocardiographic and electrocardiographic analyses. Longitudinal strain, a sensitive parameter for detecting subclinical myocardial dysfunction, was significantly reduced in the Free TE-treated group, suggesting early myocardial impairment potentially due to fibrosis, cytotoxicity, or inflammation [18]. Additionally, the marked reduction of the EF% reinforces the presence of systolic dysfunction. These mechanical impairments were further supported by ECG changes, including QRS prolongation and disappearance of the J wave. Such alterations are commonly associated with disrupted conduction pathways, repolarization abnormalities, and myocardial remodeling [37,38,39].

Mechanistically, J wave suppression may be attributed to the altered function of cardiac ion channels. TE exposure may interfere with transient outward potassium, sodium, and calcium currents, either by modulating their expression or impairing channel function [40,41]. Structural remodeling, such as fibrotic or ischemic changes, could also affect early repolarization patterns, further contributing to J wave disappearance [41]. QRS prolongation, in turn, is indicative of delayed ventricular depolarization, possibly due to conduction block, interstitial fibrosis, or cardiomyocyte damage [42,43].

Importantly, none of these adverse cardiac effects were observed in the Lip-TE-treated group, further supporting the cardioprotective potential of the liposomal encapsulation. The absence of significant reductions of the strain or EF%, combined with the preservation of QRS and J wave morphology, suggests that Lip-TE treatment effectively prevents the electrophysiological and mechanical deterioration typically associated with TE cardiotoxicity. This protective effect likely stems from improved biodistribution and reduced myocardial exposure, limiting direct toxic interactions.

RNA sequencing revealed distinct gene expression profiles among treatment groups, with the highest number of differentially expressed genes (DEGs) detected between Free TE and Lip-Blank (Figure 8, Table 4). DESeq2, which accounts for dispersion shrinkage in variable datasets [44], identified 79, 17, and 14 DEGs in the comparisons Free TE vs. Lip-Blank, Lip-TE vs. Free TE, and Lip-TE vs. Lip-Blank, respectively. Among the DEGs, *Nr1d1*, associated with vascular remodeling [23], was significantly upregulated in the Free TE group compared to the Lip-TE and Lip-Blank groups (Figure 9A). Similarly, genes linked to extracellular matrix remodeling, such as *Col4a1* and *Pxdn*, were more highly expressed in Free TE hearts, with Lip-TE showing expression levels similar to the control (Figure 9B). These results suggest that liposomal encapsulation of TE mitigates transcriptional responses related to cardiac stress and remodeling.

Collectively, the data of this study indicate that Lip-TE treatment provides superior cardioprotection and a superior pharmacokinetic and biodistribution profile compared to Free TE treatment. By reducing systemic peak concentrations and extending circulation time, liposomal encapsulation may serve as a viable strategy for enhancing the safety and efficacy of antimonial therapies. These findings support the continued development of liposomal Sb-based formulations for improved clinical outcomes.

## 5. Conclusions

The Lip-TE formulation demonstrated favorable physicochemical properties and significantly improved pharmacokinetic and biodistribution profiles compared to Free TE. These enhancements led to prolonged systemic exposure, targeted organ accumulation, and notably reduced cardiac uptake. Functionally, Lip-TE treatment preserved myocardial mechanics and electrical conduction, preventing the cardiotoxic effects observed with Free TE treatment. Transcriptomic data further supported a lower biological impact of the Lip-TE treatment at the reduced gene expression level. Collectively, these findings indicate that Lip-TE treatment is a safer and more effective strategy for antimonial therapy, supporting its continued preclinical development for visceral leishmaniasis.

## Figures and Tables

**Table 1:** Thermal program used for the determination of Sb in different organs and plasma by GFAAS.

Stage	Temperature (°C)			Argon Flow (L/min)
	Liver Time (s)	Heart/Spleen/ Kidneys Time (s)	Plasma Time (s)	Liver/Heart/Spleen/ Kidneys/Plasma
Pre-drying	85, 95 (10, 25)	85, 95 (10, 25)	85, 95 (10, 25)	0.3
Drying	120 (30)	120 (30, 10)	120 (30)	0.3
Pyrolysis	350 (35)	350 (35, 20)	350 (30)	0.3
Pyrolysis	750 (10, 5.5)	750 (10, 5.5)	750 (10, 2.5)	0.3
Atomization	2000 (0.7, 2)	2000 (0.7, 2)	2000 (0.7, 2)	0
Cleaning	2000 (3.1)	2000 (3.3)	2000 (2)	0.3

**Table 2:** Chemical and physicochemical characterization of Lip-TE and Lip-Blank.

Group	Size (nm)	Polydispersity Index (PDI)	Zeta Potential (mV)	Encapsulation Content (g/L)
Lip-TE	205.1 ± 4.5	0.284 ± 0.004	+17.0 ± 0.3	2.22 ± 0.38
Lip-Blank	137.5 ± 0.8	0.180 ± 0.020	+17.0 ± 0.6	-

Data are expressed by the mean (n = 3) ± standard deviation.

**Table 3:** Pharmacokinetics parameters obtained after administration of Free TE and Lip-TE treatments in female BALB/c mice.

Parameter	Free TE	Lip-TE
t_1/2_ (min)	177.5 ± 30.8	461.9 ± 76.9 ^a^
C_max_ (mg/L)	1.7 ± 0.3	1.3 ± 0.2
AUC_0–t_ (mg/L·min)	110.3 ± 8.1	213.9 ± 29.2 ^a^
AUC_0–inf_ (mg/L·min)	210.5 ± 24.1	675.5 ± 50.8 ^a^
MRT_0–inf_ (min)	253.0 ± 40.3	296.4 ± 43.1
V_z_ (mg/kg)/(mg/L)	13.3 ± 2.8	8.7 ± 1.3
Cl (mg/kg)/(mg/L)/min	0.04 ± 0.003	0.01 ± 0.001 ^a^

Note: Results are expressed as mean ± SEM (n = 7). t_1/2_: elimination half-life; C_max_: maximum concentration in plasma; AUC: area under the concentration–time curve; MRT: mean residence time; V_z_: apparent volume of distribution during the terminal phase; Cl: clearance. ^a^ Represents a significant difference when comparing the Free TE treatment and Lip-TE treatment (*p* < 0.05).

**Table 4:** Number of differentially expressed genes per comparison.

Comparison	Genes PF *	EdgeR	DESeq2	Common DEGs
Lip-TE vs. Free TE	25,785	0	17	0
Lip-TE vs. Lip-Blank	30,902	0	14	0
Free TE vs. Lip-Blank	29,961	0	79	0

* Genes PF refers to the number of genes retained after filtering. EdgeR/DESeq2: Number of differentially expressed genes identified by each tool. Common DEGs: Genes commonly identified by both EdgeR and DESeq2. DEGs: Differentially expressed genes.

## Data Availability

The original contributions presented in this study are included in the article. Further inquiries can be directed to the corresponding authors.
